# Supplementary material for: Co-Delivery of Novel Synthetic TLR4 and TLR7/8 Ligands Adsorbed to Aluminum Salts Promotes Th1-Mediated Immunity against Poorly Immunogenic SARS-CoV-2 RBD
Source: Vaccines (Basel). 2023 Dec 23;12(1):21. doi: 10.3390/vaccines12010021 (PMC10818338; doi:10.3390/vaccines12010021)
Supplement: Supplementary file 1 [file vaccines-12-00021-s001.zip › vaccines-2752745-supplementary.pdf]

Supplementary Materials

Table S1. Draining LN cells were recovered from animals five days after a tertiary vac-cination with the indicated vaccine, and cultured with 10μg/ml purified recombinant Spike RBD for 72h. Supernatants were tested for IFN , IL-17A and IL-5 by multiplex ELISA. Shown are values obtained from culture of cells from individual animals, or in some cases a pool of two animals if cell numbers were insufficient, plus the mean, standard deviation, and standard error of the mean. **Figure S1.** Preliminary adsorption experiments comparing the adsorption profiles of RBD, INI-4001, and INI-2002 to AH at 1:1 and 1:2 weight ratios in 15 minutes and 30 minutes in 2% glycerin. INI-4001 and INI-2002 showed >95% adsorption to AH at 1:2 weight ratio in 30 minutes.

Table S1. Draining LN cells were recovered from animals five days after a tertiary vac-cination with the indicated vaccine, and cultured with 10μg/ml purified recombinant Spike RBD for 72h. Supernatants were tested for IFN , IL-17A and IL-5 by multiplex ELISA. Shown are values obtained from culture of cells from individual animals, or in some cases a pool of two animals if cell numbers were insufficient, plus the mean, standard deviation, and standard error of the mean.

IFNγ

Antigen	Adjuvant	Cytokine Concentration (pg/ml)								Mean	Std Dev	SEM
none	(none) 2% glycerol	1.7	0.3	0.1						0.7	0.88	0.51
1ug RBD	(none) 2% glycerol	84.5	26.2	61.7						57.5	29.37	16.96
1ug RBD	10μg INI-2002 (Aq)	432.3	342.6	393.6	761.6	541.5	179.0	1227.6	676.5	569.4	324.26	114.64
1ug RBD	10μg INI-4001 (Aq)	1207.1	4241.8	4760.1	5140.9	785.5	1062.3			2866.3	2048.87	836.45
1ug RBD	INI-2002/INI-4001 (Aq)	81.4	346.3	72.2	566.4	824.2	1880.4	520.8	754.7	630.8	576.37	203.78
1ug RBD	Alhydrogel (AH pH 7)	60.3	19.4	80.5	12.4	249.3	23.6	23.5	22.9	61.5	79.44	28.09
1ug RBD	Alhydrogel (AH pH 8.1)	10.4	32.5	69.7	12.3	14.5	8.0	40.8		26.9	22.55	8.52
1ug RBD	Adju-Phos (AP)	16.3	5.9	6.7	5.5	3.6	6.4		32.3	11.0	10.28	3.89
1ug RBD	10μg INI-2002 + AH (pH 7)	2234.8	1937.9	825.4	4871.9	2426.5	907.0	4678.3	879.6	2345.2	1627.38	575.37
1ug RBD	10μg INI-2002 + AH (pH 8.1)	2464.5	1299.2	423.7	4078.3	3803.0	1771.1	403.3	4093.5	2292.1	1561.31	552.01
1ug RBD	10μg INI-2002+AP	636.7	956.7	2240.4	467.2	268.7	2230.5	2263.6	1047.4	1263.9	849.14	300.22
1ug RBD	10μg INI-4001+AH (pH 7)	4158.8	3900.2	4033.8	3551.4	4020.8	3704.4	1250.1	650.3	3158.7	1385.94	490.00
1ug RBD	10μg INI-4001+AH (8.1)	816.9	3924.0	841.8	4189.4	3229.2	3867.0	531.3	2828.0	2528.4	1551.11	548.40
1ug RBD	10μg INI-4001+AP	4025.3	3682.3	2060.2	1881.3	3361.2	3937.1	3999.6	3954.6	3362.7	887.86	313.91
1ug RBD	INI-2002/INI-4001+AH (pH 7)	3755.9	3483.9	4314.0	3736.9	3947.1	1253.2	2395.2	4074.8	3370.1	1031.53	364.70
1ug RBD	INI-2002/INI-4001+AH (pH 8.1)	3955.7	4222.6	4222.9	4027.1	1307.2	3342.9	3817.0	1932.9	3353.5	1117.92	395.24
1ug RBD	INI-2002/INI-4001+AP	3604.0	3735.2	3956.8	3645.8	3804.6	3906.0	3663.7	4129.2	3805.7	180.86	63.94

IL-17A

Antigen	Adjuvant	Cytokine Concentration (pg/ml)								Mean	Std Dev	SEM
none	(none) 2% glycerol	0.71	0.02	0.24						0.3	0.35	0.20
1ug RBD	(none) 2% glycerol	3.23	17.90	14.72						12.0	7.72	4.45
1ug RBD	10μg INI-2002 (Aq)	1192.9	583.7	771.4	971.6	1741.0	958.6	1090.2	1117.6	1053.4	340.93	120.54
1ug RBD	10μg INI-4001 (Aq)	141.2	71.5	441.6	132.6	125.5	106.7			169.9	135.42	55.29
1ug RBD	INI-2002/INI-4001 (Aq)	322.2	925.2	571.7	1160.9	1524.3	6350.3	1754.3	2810.5	1927.4	1947.19	688.43
1ug RBD	Alhydrogel (AH pH 7)	7.18	9.74	1.30	8.28	10.29	1.98	4.64	4.67	6.0	3.40	1.20
1ug RBD	Alhydrogel (AH pH 8.1)	6.57	6.15	6.29	0.67	1.10	2.65	11.27		5.0	3.75	1.42
1ug RBD	Adju-Phos (AP)	4.23	1.21	1.26	7.84	1.11	4.91	1.01		3.1	2.65	1.00
1ug RBD	10μg INI-2002 + AH (pH 7)	1359.2	2015.4	4419.4	6026.2	1731.4	790.6	1148.3	1010.9	2312.7	1887.20	667.23
1ug RBD	10μg INI-2002 + AH (pH 8.1)	2493.1	324.4	613.5	2583.1	1234.3	1944.8	345.6	2778.3	1539.6	1038.53	367.18
1ug RBD	10μg INI-2002+AP	1881.6	1022.9	1859.2	1050.2	560.7	2046.9	1665.1	720.4	1350.9	578.54	204.55
1ug RBD	10μg INI-4001+AH (pH 7)	805.8	364.7	128.1	168.0	605.2	440.6	394.2	286.1	399.1	223.73	79.10
1ug RBD	10μg INI-4001+AH (8.1)	105.1	93.6	54.6	1282.5	113.1	262.3	65.1	134.7	263.9	416.55	147.27
1ug RBD	10μg INI-4001+AP	267.4	92.6	88.0	71.3	652.7	163.4	130.9	291.0	219.7	193.24	68.32
1ug RBD	INI-2002/INI-4001+AH (pH 7)	3101.2	2060.6	3363.2	3826.5	3726.5	468.1	793.2	3651.7	2623.9	1352.73	478.26
1ug RBD	INI-2002/INI-4001+AH (pH 8.1)	1617.6	4021.1	4483.0	2204.6	448.3	3211.6	3724.9	883.1	2574.3	1507.03	532.82
1ug RBD	INI-2002/INI-4001+AP	930.3	1358.2	2345.5	1888.0	2241.1	1713.7	3120.2	2560.8	2019.7	696.36	246.20

IL-5

Antigen	Adjuvant	Cytokine Concentration (pg/ml)								Mean	Std Dev	SEM
none	(none) 2% glycerol	0.9178	0.1627	0.7485						0.6	0.40	0.23
1ug RBD	(none) 2% glycerol	808.2	3920.7	441.0						1723.3	1911.80	1103.78
1ug RBD	10μg INI-2002 (Aq)	1400.78	578.773	2072.68	624.645	2361.74	2222.29	884.746	3326.69	1684.0	975.51	344.90
1ug RBD	10μg INI-4001 (Aq)	527.199	191.037	256.722	394.563	711.137	3886.82			994.6	1429.26	583.49
1ug RBD	INI-2002/INI-4001 (Aq)	2292.47	2078.23	197.899	1273.85	2264.07	680.803	791.212	477.55	1257.0	848.47	299.98
1ug RBD	Alhydrogel (AH pH 7)	849.927	2415.26	223.155	4385.84	397.862	317.444	1927.06	1386.85	1487.9	1415.52	500.46
1ug RBD	Alhydrogel (AH pH 8.1)	3483.73	1618.92	3090.04	1011.32	1173.93	2636.98	4151.55		2452.4	1210.87	457.67
1ug RBD	Adju-Phos (AP)	1855.55	1198.23	560.82	4121.91	1054.41	1101.72	476.514		1481.3	1250.08	472.49
1ug RBD	10μg INI-2002 + AH (pH 7)	86.8528	562.271	686.627	444.188	277.53	741.422	3334.37	700.609	854.2	1027.50	363.28
1ug RBD	10μg INI-2002 + AH (pH 8.1)	576.348	251.37	2542.03	587.237	1178.93	1639	400.931	357.735	941.7	799.35	282.61
1ug RBD	10μg INI-2002+AP	538.721	754.05	417.666	1919.34	288.372	1544.52	1005	1020.95	936.1	564.20	199.48
1ug RBD	10μg INI-4001+AH (pH 7)	126.312	3012.38	881.286	1882.38	509.014	1109.15	456.865	66.4449	1005.5	1001.78	354.18
1ug RBD	10μg INI-4001+AH (8.1)	518.541	1252.24	123.921	159.8	2993.24	376.522	1024.57	1059.24	938.5	933.84	330.16
1ug RBD	10μg INI-4001+AP	1630.86	2206.27	1891.96	450.011	1654.46	333.215	2563.14	652.857	1422.8	841.56	297.54
1ug RBD	INI-2002/INI-4001+AH (pH 7)	102.319	83.6183	245.669	118.971	326.378	38.652	30.1757	94.8825	130.1	103.18	36.48
1ug RBD	INI-2002/INI-4001+AH (pH 8.1)	100.939	397.614	198.748	196.677	88.0501	87.9701	159.745	164.07	174.2	101.11	35.75
1ug RBD	INI-2002/INI-4001+AP	33.1712	119.648	115.421	108.998	66.7254	283.608	276.638	255.932	157.5	99.31	35.11

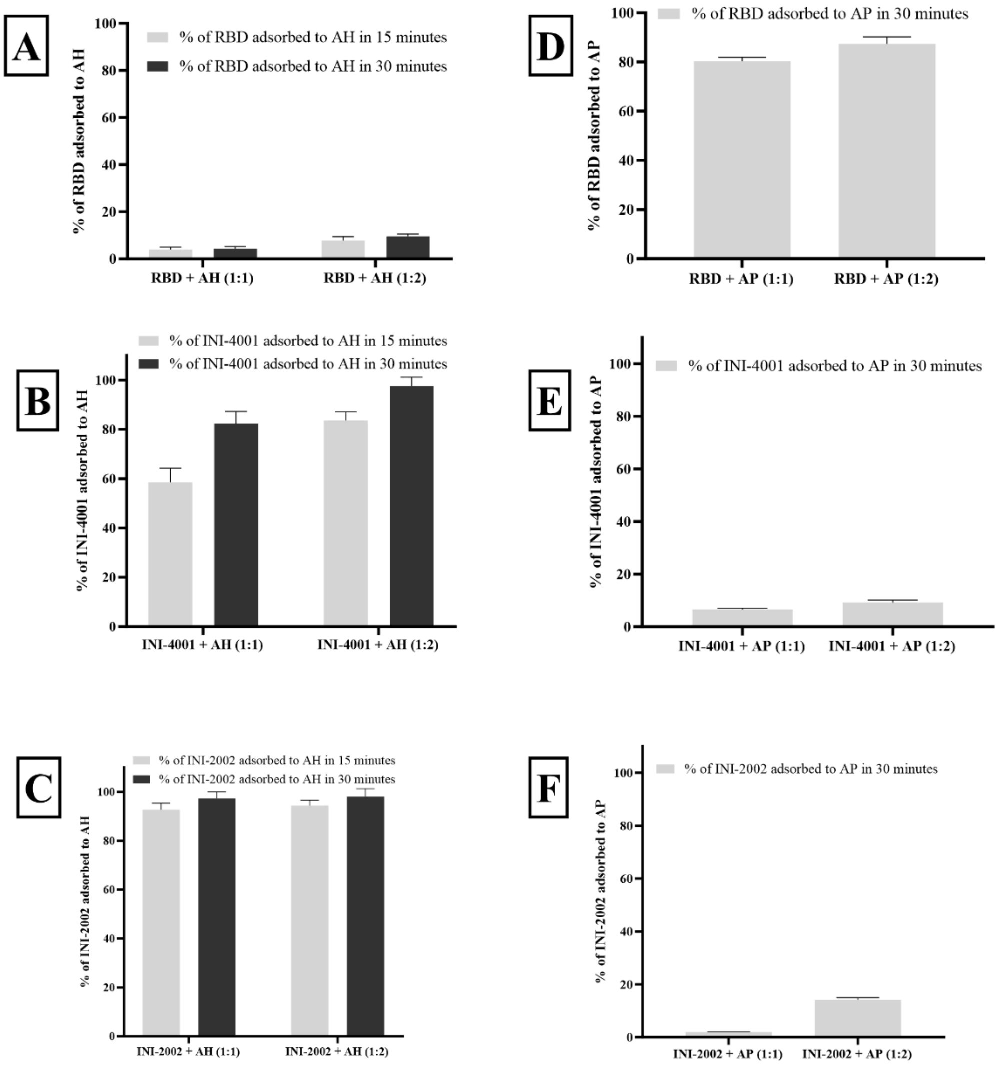

Figure S1. Preliminary adsorption experiments comparing the adsorption profiles of RBD, INI-4001, and INI-2002 to AH at 1:1 and 1:2 weight ratios in 15 minutes and 30 minutes in 2% glycerin. INI-4001 and INI-2002 showed >95% adsorption to AH at 1:2 weight ratio in 30 minutes.
